# Brain Tuberculomas: A Case Report

**DOI:** 10.5812/jjm.11252

**Published:** 2014-07-01

**Authors:** Maryam Saleh, Ali Asghar Saeedi, Ali Ali Pooran

**Affiliations:** 1 Department of Infectious Diseases, Sajad Hospital, Tehran University of Medical Sciences, Tehran, IR Iran; 2 Influenza Research Center, Pasteur Institute of Iran, Tehran, IR Iran; 3 Department of Infectious Diseases, Besat Hospital,Tehran University of Medical Sciences, Tehran, IR Iran

**Keywords:** *Mycobacterium tuberculosis*, Tuberculoma, Brain

## Abstract

**Introduction:**

An unusual incidence of tuberculosis in different parts of the body is called tuberculomas. The rate of brain tuberculosis is rare.

**Case Presentation:**

The following case of tuberculamas of the brain, presented by enhancing rings of meninges, is reported because of its rarity. It was a case of brain tuberculomas in a 15-year-old girl with primary symptoms of headache and general weakness, and no signs of primary pulmonary infection.

**Discussion:**

The subject underwent computerized tomography (CT) and magnetic resonance imaging (MRI) of the brain. Microbiological tests (acid fast bacilli smear-AFB, and culture of biopsy specimen) were applied subsequently. According to the results, the problem was diagnosed as brain tuberculomas. After operation she was completely treated with anti-TB drugs. Although brain tuberculosis is rare, it was diagnosed on the basis of histopathology and the patient's successful response to anti-tuberculous drug treatment.

## 1. Introduction

Tuberculosis (TB) is a multi-systemic infectious disease caused by different species of mycobacteria, usually *Mycobacterium tuberculosis* in humans (1). Tuberculosis of the nervous system can attack the meninges, brain, spinal cord, cranial and peripheral nerves, ears, and eyes. Brain tuberculosis results from spreading the bacteria which primarily exists in the body. A study conducted in Chile reported only one case of multiple lesions among five cases and then also recorded 33 cases of multiple tuberculomas out of 97 verified cases (2). Even in the countries where tuberculosis is common, few cases of tuberculous brain abscess have been reported. The current study showed a definite preoperative diagnosis with histological examination and radiographic findings; perhaps misdiagnosed may occur without all the findings together and it could be considered as a dilemma. Due to the possibility of concurrent infections and responsible microorganisms, it is important to evaluate all the possible related causes of the infectious diseases.

## 2. Case Presentation

In 2009, a 15-year-old girl with altered consciousness, drowsiness and abnormality in gait motor and sensation of left side extremities (with no signs of pulmonary infection) was referred to the emergency room of Sajad Hospital. She was the first child of acouple with no family relationship. She was studying in a high school with low level of sociocultural state in North of Iran. Her family did not have any symptoms of tuberculosis. There was a history of general weakness in the recent six months with internal work up and progression to neurological deficit. She could not move her right side of the body but she responded to questions on and off and obeyed the orders. She could respond to medical interrogation. She was oriented in time, place and person. Paresthesia of left extremities and neck problem was found. Cranial nerves, sensory and motor-systems were normal with no signs of meningeal irritation. Skull X-ray revealed normal.

Paraclinical and physical examinations such as Chest X-ray, brain Computerized Tomography (CT) Scan, abdomino pelvic CT scan and brain Magnetic Resonance Imaging (MRI) were applied. Microscopic examination (direct examination) of a biopsy specimen was also performed. The primary diagnosis was herpes with differential diagnosis due to involvement of parieto-temporal lobe in her CT scan. She received acyclovir and was admitted in infectious disease ward. The patient did not respond to the treatment. Multislice helical CT scan of chest, with and without contrast media, showed extensive cavitary consolidation at left upper lobe. Peribroncho vascular nodular infiltration with tree-in-bud appearance was noted at posterior and apical segments of upper and lower lobes. Pleural effusion was not observed. Mediastinal adenopathies were present in prevascular, paratracheal and azygoesophageal recess distributions. Multislicehelical CT-scan of abdominopelvic with and without contrast media found mild ascites around the liver and pelvic cavity. Multiple hypodense lesions in favor of adenopathies were noted at portahepatis celiac chain, retrocaval and precaval levels. Mild dilatation was visualized at both pelvicalyceal systems.

Histological studies demonstrated no primary focus in the lungs. Cavitation and tree-in-bud were observed in CT scan of lungs. Axial CT scan of the brain demonstrated large hypodensity area and ring-enhancing lesion in right middle cerebral artery territory with some hemorrhagic area. Edema in right hemisphere and compression on right lateral ventricle and some midline shift to left were noted. Hypodensity area in left cerebellum was observed too. All of the symptoms were compatible with those of ischemic hemorrhage or metastatic hemorrhage. The grate cerebral vein and the venous sinuses had normal caliber in Magnetic Resonance Venography (MRV). Other Organs such as liver, spleen, kidneys, gallbladder and uterus were normal.

**Figure 1.**
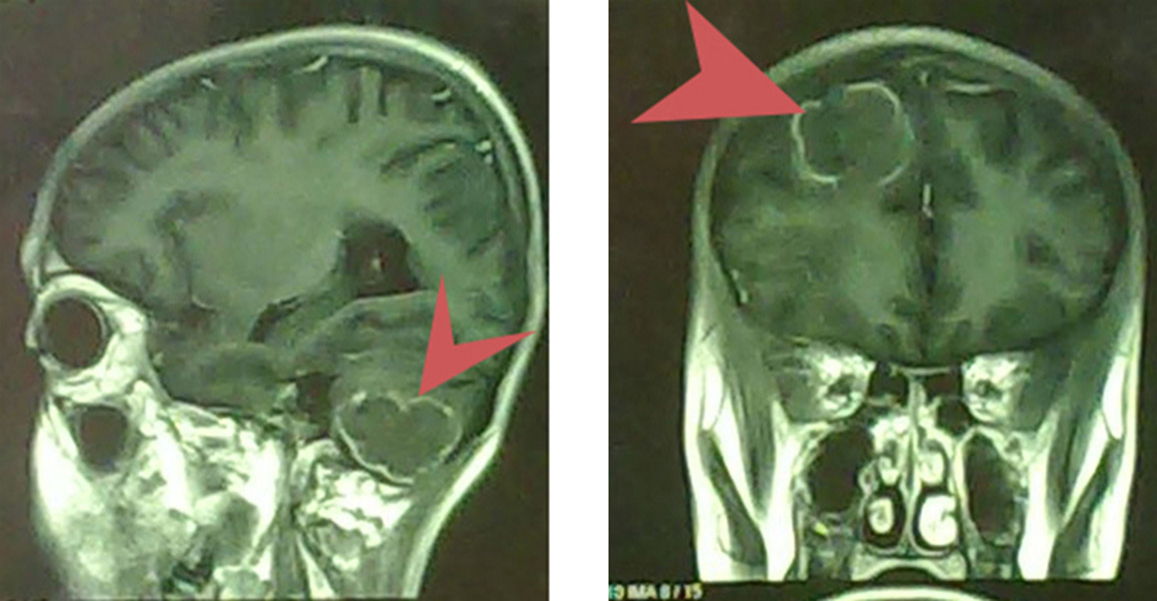
The magnetic resonance image of the brain showing enhancing nodules in deep white matter and subcortical area in the right fronto-parieto-temporal region and also left cerebellar hemisphere showing foci of hemorrhage with surrounding edema and mass effect and irregularity ring enhancement

**Figure 2.**
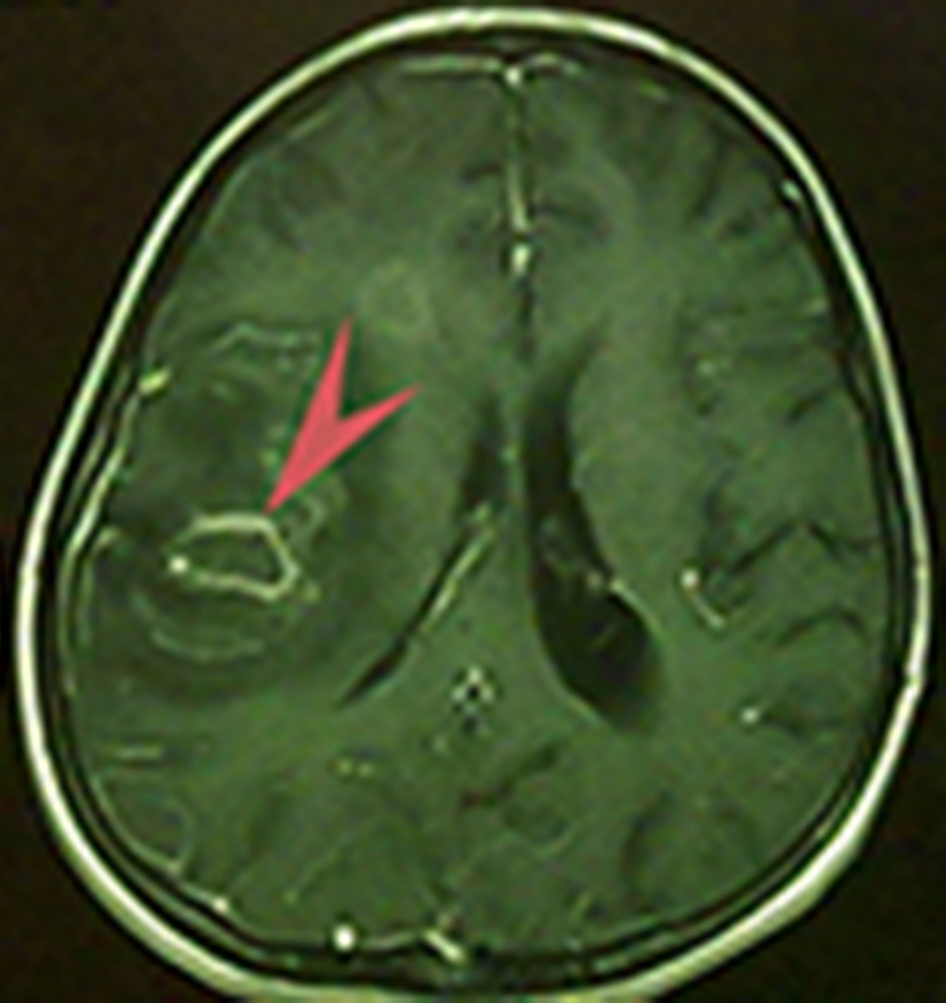
Brain MRI illustrates a separate circular mass at the top of frontal region with hemorrhage in the right fronto-parietal and left cerebellar hemisphere and air fluid level in right maxillary sinus

Magnetic resonance imaging (MRI) examination of the brain was performed with intravenous contrast, and utilizing axial T1-weighted sequences. There were multiple T1 low signal masses in the right fronot-parie to-temporal region and also left cerebellar hemisphere showing foci of hemorrhage with surrounding edema and mass effect and irregularity ring enhancement. No other abnormalities were observed (Figure 1).

brain MRI with and without gadolinium (GD) injection determined post-op changes with mass effect and hemorrhage in the right-fronto-parietal and left cerebellar hemisphere. No abnormal enhancement was reported after contrast injection. Air fluid level was observed in the right maxillary sinus. No other abnormalities were observed (Figure 2). In the physical exam, ill-defined borders were pale. 

Microscopic examination: The most sensitive tests, acid fast bacilli smear (AFB) and culture of a biopsy specimen, were performed. Puncturing viscous fluid material was taken, smears were made and stained with acid-fast stain and a number of acid-fast bacilli were observed (4^+^).

Treatment and drugs: Antibiotics: ciprofloxacin (500-750ng), ethambotol (800 ng), isoniazid (300 ng) and omeprazole (20 mg) were prescribed.

Surgery: For patients with neurological deficits, surgical intervention is recommended (3, 4). The patient underwent the brain surgery. Multiple sections were taken and studied in detail histologically. Pathological right frontal lesion specimen of the brain showed extensive areas of caseating like necrosis suggesting histological diagnosis of tuberculomas.

## 3. Discussion

Tuberculosis is still a leading cause of death among the infectious diseases. Because of the current migratory patterns, tuberculosis has become a global concern and early diagnosis of active Tuberculosis is vital for its treatment. The World Health Organization (WHO) declared tuberculosis as a global emergency in 1993. Provisional data from Health Protection Agency UK (HPA) showed that 8587 cases of tuberculosis were reported in the UK in 2010. This is a rate of 13.9 per 100000 population. In 2008, 334 patients died from tuberculosis which is 0.6 per 100000 population. These data are significantly better, compared those of1955 in which 3900 patients died from tuberculosis with mortality rate of 8.8 per 100000 population (5). In 2012 report of a case presented with prolonged nonspecific symptoms and multiple brain tuberculomas demonstrated that diagnosis of such peculiar presentations is very important (6).

Brain abscesses are tuberculomas that develop into pus-filled cavities and indicate poor defense mechanisms. They are rare and may require surgical excision (7). Tuberculomas constitute 33% of intracranial space-occupying lesions in patients in developing countries (8). Calcification may be present within the contrast enhanced ring, creating a target sign which is probably a sign of reactivation (9). Brain tuberculomas is rare and its diagnosis would be difficult and impossible because of its variety of presentation, therefore clinical findings and special tests are needed. CT was reported to have a sensitivity of 100% and specificity of 85.7% thus it indicated a need for further analysis with MRI that should be the technique of choice and/or histological diagnosis (10). The CT findings in the current case showed multiple ring enhancing lesions but finding based on CT alone is presumptive. Biopsy of the brain is the most accurate method of diagnosis in case of multiple brain tuberculomas, and the findings of the case by surgery suggested it as the final diagnosis. 

The diagnosis should be supported by findings such as history of fever, high ESR, positive tuberculin test and positive response to anti-tuberculosis treatment (11). MRI is reportedly superior to CT for diagnosis of brain tuberculomas. In conclusion, CT scan and MRI provided essential information that aids the diagnosis of brain tuberculomas (10), therefore suspected TB cases should do the diagnostic tests. CT scans of the lungs in TB patients indicating the centrilobular nodules and branching linear opacities of similar caliber originating from a single stalk (the tree-in-bud pattern) were primarily a sign of abnormalities (12). However, it can be concomitant with other infectious diseases, immunologic disorders, congenital disorders, neoplasm, and diseases with idiopathic causes (13, 14).

Clusters of small nodules may be observed in pulmonary tuberculosis and pulmonary sarcoidosis, and may be a CT finding of active pulmonary tuberculosis. But what distinguishes between the two is association of a single cluster of small nodules, clusters of small nodules in the superior segment of the lower lobe, or clusters of small nodules with tree-in-bud lesions in pulmonary tuberculosis not associated with lymphadenopathy (15). 

In conclusion, the current case made complete recovery on anti- tuberculosis treatment as the last treatment. The frequency of brain tuberculosis depends on the site involved. Possible side effects of anti-TB drugs of patients should be clinically evaluated at least monthly. Patients usually do not need the follow-up after completion of therapy if signs or symptoms do not recur. In the current study the patient was followed up for three years, and CT after one year showed complete disappearance of all tuberculomas. Microscopic examination and mycobacterial culture fitted into her recovery as well.
